# Increased Geriatric Treatment Frequency Improves Mobility and Secondary Fracture Prevention in Older Adult Hip Fracture Patients—An Observational Cohort Study of 23,828 Patients from the Registry for Geriatric Trauma (ATR-DGU)

**DOI:** 10.3390/jcm10235489

**Published:** 2021-11-23

**Authors:** Johannes Gleich, Evi Fleischhacker, Katherine Rascher, Thomas Friess, Christian Kammerlander, Wolfgang Böcker, Benjamin Bücking, Ulrich Liener, Michael Drey, Christine Höfer, Carl Neuerburg

**Affiliations:** 1 Department of Orthopaedics and Trauma Surgery, Musculoskeletal University Center Munich (MUM), University Hospital, LMU Munich, 81377 Munich, Germany; Johannes.Gleich@med.uni-muenchen.de (J.G.); Evi.Fleischhacker@med.uni-muenchen.de (E.F.); Wolfgang.Boecker@med.uni-muenchen.de (W.B.); 2 AUC—Academy for Trauma Surgery, 80538 Munich, Germany; Katherine.Rascher@auc-online.de (K.R.); Thomas.Friess@auc-online.de (T.F.); Christine.Hoefer@auc-online.de (C.H.); 3 AUVA Traumahospital Styria, 8020 Graz, Austria; UOA@auva.at; 4 Center for Orthopedics and Trauma Surgery, DRK-Kliniken Nordhessen, 34121 Kassel, Germany; benjamin.buecking@web.de; 5 Department of Orthopedics and Trauma Surgery, Marienhospital, 70199 Stuttgart, Germany; Ulrich.Liener@vinzenz.de; 6 Department of Medicine IV, University Hospital, LMU Munich, 80336 Munich, Germany; Michael.Drey@med.uni-muenchen.de

**Keywords:** hip fracture, orthogeriatric care, fragility fracture, interdisciplinary treatment, osteoporosis, registry

## Abstract

Interdisciplinary orthogeriatric care of older adult hip fracture patients is of growing importance due to an ageing population, yet there is ongoing disagreement about the most effective model of care. This study aimed to compare different forms of orthogeriatric treatment, with focus on their impact on postoperative mobilization, mobility and secondary fracture prevention. In this observational cohort study, patients aged 70 years and older with a proximal femur fracture requiring surgery, were included from 1 January 2016 to 31 December 2019. Data were recorded from hospital stay to 120-day follow-up in the Registry for Geriatric Trauma (ATR-DGU), a specific designed registry for older adult hip fracture patients. Of 23,828 included patients from 95 different hospitals, 72% were female, median age was 85 (IQR 80–89) years. Increased involvement of geriatricians had a significant impact on mobilization on the first day (OR 1.1, CI 1.1–1.2) and mobility seven days after surgery (OR 1.1, CI 1.1–1.2), initiation of an osteoporosis treatment during in-hospital stay (OR 2.5, CI 2.4–2.7) and of an early complex geriatric rehabilitation treatment (OR 1.3, CI 1.2–1.4). These findings were persistent after 120 days of follow-up. Interdisciplinary treatment of orthogeriatric patients is beneficial and especially during in-patient stay increased involvement of geriatricians is decisive for early mobilization, mobility and initiation of osteoporosis treatment. Standardized treatment pathways in certified geriatric trauma departments with structured data collection in specific registries improve outcome monitoring and interpretation.

## 1. Introduction

Interdisciplinary orthogeriatric care revealed encouraging results for the treatment of older adult patients suffering a proximal femur fracture with reduction of peri-/postoperative complications and preservation of activities of daily living and independency [1,2,3]. Due to the sudden loss of function and high prevalence of comorbidities, these patients are at risk of poor recovery, expressed by a one-year mortality up to 20% [4,5,6]. Consequently, health loss in hip fracture patients expressed in DALY (disability adjusted life years) is expected to double from 2020 to 2040, while the socioeconomic costs are estimated to increase by 65% [7]. Various parameters seem to play a crucial role in the recovery process of orthogeriatric patients, such as previous walking ability or the occurrence of complications like decubitus or delirium [8]. Early mobilization with full weight-bearing is of major importance in the postoperative course, as weight-bearing restrictions reduce mobility and early mobilization increases odds for discharge by 30-day postoperatively [9,10]. Moreover, the initiation of an osteoporosis therapy showed significant impact on mortality and secondary fracture prevention in patients with osteoporosis related fractures [11]. The implementation of orthogeriatric care varies from one country and from one hospital to the other, the impact of divergent extents of geriatricians’ involvement remains unclear. Specialized registries could provide deeper understanding of essential treatment elements and evaluate the impact of interdisciplinary care [12]. Various established registries have been analyzed so far, such as the Australian & New Zealand Hip Fracture Registry, the Danish Multidisciplinary Hip Fracture Registry (DMHFR), the Spanish National Hip Fracture Registry (RNFC) and the British National Hip Fracture Database (NHFD) [13,14,15,16]. Besides variable knowledge on the choice of implants, different surgical approaches or time-to-surgery, heterogeneous inclusion criteria are common in these registries. Only little information on the individual patient is documented with regards to comorbidities, mobilization, secondary fracture prevention and extent of involvement of geriatricians. Analysis of the multicenter Registry for Geriatric Trauma (ATR-DGU), established by the German Trauma Society (DGU), could significantly contribute to a better understanding of treatment results. Inclusion is only performed by previously certified departments (Alters Trauma Zentrum DGU^®^ (ATZ-DGU)), assuring standardized interdisciplinary treatment based on a predetermined criteria catalogue and audits as well as standardized data collection, and therefore leading to a comprehensive description of the included patients [17]. In the present study we hypothesized that the extent of involvement of geriatricians affects the treatment of older adult hip fracture patients in an orthogeriatric care setting, regarding early mobilization, mobility and osteoporosis treatment.

## 2. Materials and Methods

The Registry for Geriatric Trauma (ATR-DGU) is a multicenter database founded by the German Trauma Society (DGU). Since 2016, all hospitals certified as AltersTraumaZentrum DGU^®^ have been required to enter their patients’ characteristics into this database. In total, about 100 hospitals have been involved, most of them located in Germany, with a few also in Switzerland and Austria. From 2016 through 2019 almost 25,000 cases were documented in the ATR-DGU. The data of all patients aged 70 years and older, who suffered a fracture of the proximal femur requiring surgery are entered into the database. The initial data collection is based on standardized questionnaires that cover five phases of hospitalization: admission, preoperative phase, surgery, postoperative phase, discharge. The questionnaires were developed together with the Fragility Fracture Network (FFN), taking into account experiences from the “National Hip Fracture Database” of England and Wales, and the “Australian and New Zealand Hip Fracture Registry”, to allow international comparison. They collect parameters such as walking ability before the accident, a pre-existing level of care, intake of anticoagulants and osteoporosis medication upon admission, a geriatric assessment (including the ISAR score, a six-item screening tool for elderly patients in the emergency department, collecting data about functional dependence, recent hospitalization, impaired memory and polypharmacy [18]), and general information on the age and sex of the patient. The surgery phase is represented by information on fracture configuration, surgical and anesthesia procedures and ASA-Classification (American Society of Anesthesiologists). For the postoperative phase, walking ability, initiation of an osteoporosis treatment and interdisciplinary treatment by a geriatrician during the first seven postoperative days are documented. Information about the discharge location is also collected (home, rehabilitation clinic, nursing home etc.). At two follow-up points (7 and 120 days after surgery), walking ability, status of osteoporosis treatment, re-operation rate and patients’ whereabouts are assessed. Participating hospitals have to meet various criteria for certification as ATZ-DGU: interdisciplinary treatment by trauma surgeons and geriatricians, ensured geriatric treatment frequency (ranging from consultation based at least twice a week to continuous collaborative treatment), standard operating procedures for surgical treatment/pain management/mobilization/delirium assessment and prevention/osteoporosis assessment/discharge management and many more. Regarding mobilization, each patient received daily sessions of physiotherapy, while parts of this were performed as group therapy if possible. The primary objective was the earliest possible mobilization out of bed with full weight-bearing. To prevent or treat postoperative delirium, a clearly structured daily schedule was given, which starts with activating body care with assistance in the morning, followed by shared breakfast with other patients (if possible) and the first physiotherapy session, then lunch and second session, ending with supper. Regular interdisciplinary team meetings including surgeons, geriatricians, nurses, physiotherapists, social workers and others also addressed the individual patients’ needs.

Patients were divided into two groups, depending on the visit frequency of the geriatrician (at least/more than twice a week) for this study. The visit frequency was queried by default during data collection and entry for ATR-DGU and therefore was used for definition of the two groups in this study. Records from hospitals with an interdisciplinary treatment frequency less than twice a week were excluded. In hospitals with a visit frequency more than twice a week, no weekend or out of hours service was delivered routinely. No distinction was made between different fracture types and all fractures of the femoral neck, trochanteric fractures and periprosthetic fractures of the proximal femur were included. The infrastructure for data entry, data management and data analysis is provided and maintained by the AUC—Academy for Trauma Surgery (AUC), an institution affiliated to the German Trauma Society (DGU). The scientific leadership is incumbent on the Working Committee on Geriatric Trauma Registry (AK ATR) of the German Trauma Society (DGU). Following a peer-review procedure, scientific data analysis is approved according to a peer review procedure laid down in the publication guideline of ATR-DGU. The present study was approved with project number ATR-2020-005. This study followed the Strengthening the Reporting of Observational Studies in Epidemiology (STROBE) reporting guideline for cohort studies. Data analysis received approval from the Ethics Committee of the medical faculty of the LMU Munich, Munich, Germany (Reg. No. 234-16) and from the Ethics Committee of the medical faculty of the Philipps-University, Marburg, Germany (AZ 46/16). Data are available from the Registry for Geriatric Trauma (ATR-DGU) after approval by the Working Committee on Geriatric Trauma Registry (AK ATR) of the German Trauma Society (DGU). 

For descriptive analyses, categorical data were presented as counts and percentages, continuous variables as median with interquartile range (IQR). Some patients had missing data for individual parameters; therefore, each analysis shows the total number of patients that were analyzed. Comparisons between the two groups were made using Χ^2^-test for categorical variables and the Mann Whitney U-Test for continuous variables. Linear and logistic regression models were used to examine the impact of geriatric treatment frequency on a range of outcomes 7 and 120 days after surgery. All multivariate analyses were adjusted for age, gender and ASA score. Results are reported as regression coefficient (ß) for linear regression and Odds Ratios (OR) for logistic regression along with their 95%-confidence intervals (CI). Differences were considered statistically significant when *p* < 0.05. All calculations were performed using statistics software R v. 4.0.2 (Foundation for Statistical Computing, Vienna, Austria).

## 3. Results

**Figure 1:**
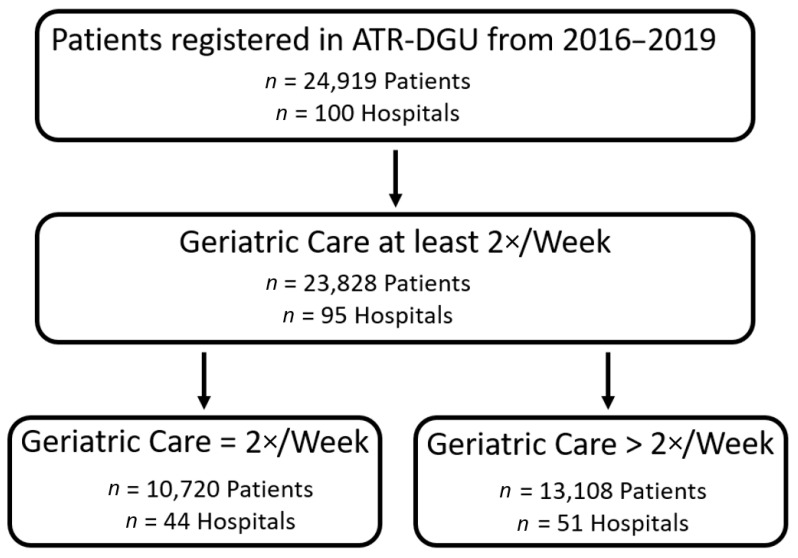
Flow chart presenting inclusion process during study period.

In total, 23,828 patients from 95 hospitals were considered for final analysis (Figure 1). Women represented 72% of the study population and the median age was 85 (80–89) years. Regarding to the ASA-Classification, almost 77% of the patients had a severe systemic disease (defined by an ASA-Classification ≥3); prior to fracture, only 34% could walk unaided and 80% had no existing osteoporosis treatment. A geriatric treatment frequency of 2 times per week was observed in 45% and more than 2 times per week in 55% of the patients (Table 1). Baseline data showed slight differences between the groups regarding age, ASA score, walking ability/place of residence and osteoporosis treatment pre-fracture; as mentioned above, these significances could be attributed to the large sample size and should be interpreted with caution (Table 1). An increased frequency of geriatric treatment showed a significant impact on mobilization on the first day and mobility seven days after surgery, initiation of an osteoporosis treatment during in-hospital stay and of an early complex geriatric rehabilitation treatment (Table 2). These findings were persistent after 120 days of follow-up with increased walking ability and increased odds for secondary fracture prevention regarding osteoporosis treatment for patients treated in hospitals with intensified geriatric involvement (Table 3). Odds for in-house mortality and mortality during follow-up showed no significant difference between the groups; although time to surgery was slightly longer in hospitals with increased geriatric treatment frequency, no significant influence was observed in linear regression analysis. Multivariate regression analysis also demonstrated no significant influence of geriatric care frequency on place of residence after discharge from the hospital, but after 120 days of follow-up. Here, living at home was less likely in the group with geriatric care more than twice a week (Table 3).

## 4. Discussion

Older adult hip fracture patients often present with a variety of age associated physiological changes and comorbidities. Interdisciplinary treatment approaches aim to address their complex needs. In order to improve outcome monitoring and patient safety, and to identify the numerous factors affecting treatment results in these patients, different registries from regional to international levels have been established in orthopedic research [19]. Despite these efforts, Newgard CD et al. concluded, that trauma registries in their current form are ineffective in capturing, tracking and evaluating injured older adults and demanded a different approach to assess the quality of care with registries [20].

In the present study, analysis of the Geriatric Trauma Registry (ATR-DGU) provided new insights on orthogeriatric care of older adult hip fracture patients and data acquisition in a specific designed registry. An established certification process for participating hospitals was mandatory to provide patients data, which reduced the risk of heterogeneity and assured a standardized interdisciplinary treatment [17]. Only hip fracture patients >70 years of age are included in the ATR-DGU; the observed majority of patients classified ASA 3, the variety of comorbidities and huge pre-hospital dependence on walking aids underlines the specific focus on orthogeriatric patients in this registry. Due to the certification criteria no reference group without geriatric co-treatment (usual orthopedic care) is assessed within the registry; to the best of our knowledge, therefore this is the first study to compare different extents of interdisciplinary orthogeriatric care in older adult hip fracture patients.

The first key finding points out that early mobilization in an interdisciplinary care setting of older adult hip fracture patients is improved by increased geriatric involvement. Other health services report mobilization rates from 43% to 79% in the first 36–48 h after hip fracture surgery in an usual care setting [10,21]. This is of particular importance, as short-term muscle disuse could lead to muscle atrophy and sarcopenia, which accumulates throughout an individuals´ lifespan [22]. A comparative study between younger and older individuals demonstrated that aging impairs the recovery in mechanical muscle function following four days of disuse, and that older patients will not achieve complete recovery from muscle disuse, which bears the risk to enter a circle of frailty [23,24]. Moreover, initiation of early complex geriatric care (specific physical and occupational therapy with focus on independence in activities of daily living) and mobility seven days after surgery were beneficially influenced by increased geriatric treatment. As physical function at discharge was identified as modifiable prognostic factor for long-term physical function after hip fracture, this effort is worth making and was verified by better walking ability after 120 days of follow-up in patients with increased geriatric treatment frequency [25].

Furthermore, our results illustrate that in this multidisciplinary setting treatment initiation of an underlying osteoporosis is particularly influenced by the treatment frequency of the geriatrician. Subsequently diagnostics and start of treatment are essential, as hip fracture patients are at twofold risk suffering a secondary hip fracture and adherence to osteoporosis therapy is associated with significant reduction of secondary fracture risk and mortality [11,26]. Studies on specific osteoanabolic therapy of hip fracture patients already reported improved functional performance and reduced hip pain [27]. Regarding the different forms of orthogeriatric care our findings suggest that the extent of involvement of geriatricians is also decisive for secondary fracture prevention. Persistent higher odds for osteoporosis therapy after 120 days of follow-up confirmed these findings. This is in line with previous studies, which demonstrated that differences in infrastructure, logistics and clinical practice in the management of trauma patients have significant impact on patients outcome [28]. Interestingly, no influence of geriatric treatment frequency was observed on in-house mortality and after 120 days of follow-up. This differs from data reported in other hip fracture registries: the analysis of a multidisciplinary care approach in comparison to usual orthopedic care with a similar number of patients observed significantly reduced 30-day mortality in patients treated with orthogeriatric care [29]. Neuburger et al. also demonstrated in contrast to these findings a beneficial effect of increased orthogeriatrican hours per patient on 30-day mortality after analysis of 196,401 patients in England. This strengthens the hypothesis that orthogeriatric treatment per se is beneficial, but also the ‘dose per week’ has an impact on patients’ outcomes [30]. As the present study is an observational study on different extents of interdisciplinary orthogeriatric treatment only (no “usual orthopedic care” as reference) and standardized treatment protocols were used in all hospitals, this could have improved overall treatment results and therefore no difference was observed. The observed lower odds for living at home after increased geriatric treatment at the 120-day follow-up may be explained through intensified efforts to improve patients´ living conditions beyond their in-hospital stay and therefore arranging additional help in the form of assisted living/changes in their place of residence. The findings of this study may not only be explained by the increased frequency of geriatric treatment alone. Furthermore, the increased amount of interdisciplinary treatment with specialized and sensitized nurses and physiotherapists may have a positive impact on daily mobilization and subsequent walking ability; the geriatrician may share their knowledge with the attending physician/surgeons in interdisciplinary ward rounds, so they can continue orthogeriatric treatment even in their absence. These effects on the treating team should be evaluated in further studies.

Some limitations of this registry analysis have to be considered. Only complete records for each specific research question were used, therefore number of included patients vary. This could have introduced selection bias. Due to the design of the registry and the standardized data acquisition, no information about individual comorbidities or additional scores besides ASA and ISAR score were recorded. As walking ability was one of the main outcome parameters, this could have affected these results. Despite that, regaining mobility after a proximal femur fracture depends on various factors, the beneficial impact of early mobilization, physiotherapy and specific orthogeriatric treatment approaches was already shown [9,10]. Some variables were only dichotomously recorded (yes/no), which only allows a basic assessment of these parameters, but no further evaluation (e.g., postoperative degree of ambulation). Outcome parameters were also only assessed during the in-patient stay and after 120 days, which might over- or under-estimate some findings. As not all participating hospitals provide data for 120-day follow-up and therefore these data are only available for a smaller group of the registry population, these results have to be interpreted with caution.

## 5. Conclusions

This study investigates a specifically designed registry to assess orthogeriatric treatment of older adult hip fracture patients, only including datasets from previously certified hospitals, ensuring standardized treatment and data collection. Our findings demonstrate that interdisciplinary orthogeriatric treatment of these patients is beneficial for early mobilization, mobility and initiation of osteoporosis treatment and that increased geriatric involvement affects their course from in-patient stay to follow-up.

## Figures and Tables

**Table 1:** Baseline characteristics of older adults with geriatric treatment more than two times/two times per week following hip fracture surgery.

	Geriatric Treatment >2×/Week	Geriatric Treatment 2×/Week	*p*-Value
**Total Patients**	*n* = 13,108	*n* = 10,720	
**Age** (years)	(*n* = 12,979)	(*n* = 10,623)	**0.016**
median (IQR)	85 (80; 89)	84 (80; 89)	
**Sex**	(*n* = 13,082)	(*n* = 10,687)	0.325
Female	9503 (72.6%)	7701 (72.1%)	
**ASA**	(*n* = 12,909)	(*n* = 10,566)	**0.009**
1	147 (1.1%)	113 (1.1%)	
2	2780 (21.5%)	2454 (23.2%)	
3	8943 (69.3%)	7240 (68.5%)	
4	1031 (8%)	751 (7.1%)	
5	8 (0.1%)	8 (0.1%)	
**ISAR Score**	(*n* = 8220)	(*n* = 7260)	**0.001**
0	773 (5.0%)	493 (3.2%)	
1	1036 (6.7%)	830 (5.4%)	
2	1833 (11.8%)	1507 (9.7%)	
3	1915 (12.4%)	1887 (12.2%)	
4	1698 (11.0%)	1549 (10.0%)	
5	748 (4.8%)	728 (4.7%)	
6	217 (1.4%)	266 (1.7%)	
**Walking ability pre-fracture**	(*n* = 12,091)	(*n* = 10,035)	**0.002**
Without aids	4214 (34.9%)	3273 (32.6%)	
With one crutch/cane	1587 (13.1%)	1279 (12.7%)	
With 2 crutches/walker	3841 (31.8%)	3368 (33.6%)	
Only at home	2041 (16.9%)	1787 (17.8%)	
none	408 (3.4%)	328 (3.3%)	
**Place of residence pre-fracture**	(*n* = 3420)	(*n* = 2709)	**<0.001**
At home	3039 (88.9%)	2409 (88.9%)	
Nursing Home	198 (5.8%)	107 (3.9%)	
Hospital (Inpatient fracture)	78 (2.3%)	57 (2.1%)	
Other	105 (3.1%)	136 (5%)	
**Type of fracture**	(*n* = 13,071)	(*n* = 10,683)	0.135
femoral neck	5602 (42.9%)	4674 (43.8%)	
pertrochanteric	6024 (46.2%)	4874 (45.6%)	
subtrochanteric	540 (4.1%)	393 (3.7%)	
periprosthetic	709 (5.4%)	589 (5.5%)	
other	196 (1.5%)	153 (1.4%)	
**Osteoporosis treatment pre-fracture**	(*n* = 12,564)	(*n* = 10,375)	**<0.001**
Yes	2886 (23.0%)	1787 (17.2%)	
**Mobilization 1 day after surgery**	(*n* = 12,883)	(*n* = 10,609)	0.078
Yes	10,355 (80.4%)	8428 (79.4%)	
**Walking ability 7 days after surgery**	(*n* = 12,585)	(*n* = 10,297)	**<0.001**
No mobility	9699 (77.1%)	8201 (79.6%)	
Able to walk (with/without assistance)	2886 (22.9%)	2096 (20.4%)	
**Osteoporosis treatment**	(*n* = 13,031)	(*n* = 10,672)	**<0.001**
**7 days after surgery**			
Yes	9622 (73.8%)	5623 (52.7%)	
**Initiation of early**	(*n* = 11,089)	(*n* = 9352)	**<0.001**
**complex geriatric care**			
Yes	7150 (64.5%)	5428 (58.0%)	
**Time to surgery**	(*n* = 12,979)	(*n* = 10,612)	**0.002**
Median (IQR) in hours	18.1 (7.75; 26.7)	17.8 (7.0; 24.8)	
**Revision surgery**	(*n* = 13,090)	(*n* = 10,709)	**0.0089**
**Yes**	483 (3.7%)	328 (3.1%)	
**Mortality**	(*n* = 12,733/5392)	(*n* = 10,395/3249)	**0.0643**
inpatient	737 (5.8%)	543(5.2%)	**0.0639**
120 day follow-up	625 (11.6%)	365 (11.2%)	
**Discharge Location**	(*n* = 12,966)	(*n* = 10,645)	**<0.0001**
Home	2880 (22.2%)	2357 (22.1%)	
Nursing Home	3700 (28.5%)	2532 (23.8%)	
Rehabilitation clinic	5164 (39.9%)	4904 (46.2)	
Other Hospital	233 (1.8%)	250 (2.3%)	
Other hospital ward	44 (0.3%)	28 (0.3%)	
Other	208 (1.6%)	31 (0.3%)	
Died in-house	737 (5.7%)	543 (5.1%)	

Abbreviations: ASA, American Society of Anesthesiologists; ISAR Score, Identification of seniors at risk. Mann-Whitney *U*-Test was used for continuous variables, chi-squared test for discrete variables. Bold font indicates statistical significance.

**Table 2:** Multivariable logistic and linear regression analysis of the impact of increased geriatric treatment frequency on various outcomes during the initial hospital stay.

Impact of Geriatric Treatment Frequency on	*N*	OR	95%-CI	*p*-Value
**Mobilization 1 day after surgery**	23,383	1.07	(1.00; 1.14)	**0.040**
**Walking ability 7 days after surgery**	22,768	1.14	(1.07; 1.22)	**<0.001**
**Osteoporosis treatment 7 days after surgery**	22,735	2.54	(2.40; 2.70]	**<0.001**
**Initiation of early complex geriatric care**	20,422	1.32	(1.24; 1.39)	**<0.001**
**Discharge to home**	21,496	1.05	(0.98; 1.11)	0.162
**Inpatient mortality**	23,615	1.09	(0.96; 1.22)	0.167
	*N*	β		
**Time to surgery (hours)**	23,468	−0.03	(−0.84; 0.77)	0.934

Reference: Geriatric care two times per week; all models were adjusted for age, sex and ASA Score; model also adjusted for “osteoporosis treatment before fracture”. Bold font indicates statistical significance.

**Table 3:** Multivariable logistic regression analysis of the impact of increased geriatric treatment frequency on various outcomes after 120 days of follow-up.

Impact of Geriatric Treatment Frequency on	*N*	OR	95%-CI	*p*-Value
**Walking ability**	7590	1.10	(1.00; 1.21)	**0.047**
**Osteoporosis treatment 120 days after treatment**	5351	1.68	(1.50; 1.90)	**<0.001**
**Living at home**	7415	0.73	(0.66; 0.82)	**<0.001**
**Death within follow-up**	8571	1.02	(0.88; 1.17)	0.527

Reference: Geriatric care two times per week; all models were adjusted for age, sex and ASA Score; model also adjusted for “osteoporosis treatment before fracture”. Bold font indicates statistical significance.

## Data Availability

Restrictions apply to the availability of these data. Data was obtained from the Registry for Geriatric Trauma (ATR-DGU) and are available from the Academy for Trauma Surgery (AUC) with the permission of the Working Committee on Geriatric Trauma Registry (AK ATR) of the German Trauma Society (DGU).
